# Study on biodegradation mechanism of *Fusarium solani* NK-NH1 on the hull wood of the Nanhai No. 1 shipwreck

**DOI:** 10.3389/fmicb.2024.1382653

**Published:** 2024-05-30

**Authors:** Yu Wang, Yeqing Han, Naisheng Li, Cen Wang, Kaixuan Ma, Xinduo Huang, Jing Du, Hong Guo, Jiao Pan

**Affiliations:** ^1^Key Laboratory of Archaeomaterials and Conservation, Ministry of Education, University of Science and Technology Beijing, Beijing, China; ^2^Institute for Cultural Heritage and History of Science and Technology, University of Science and Technology Beijing, Beijing, China; ^3^Department of Microbiology, College of Life Sciences, Nankai University, Tianjin, China; ^4^National Centre for Archaeology, Beijing, China

**Keywords:** the Nanhai No. 1 shipwreck, *Fusarium solani*, biodegradation mechanism, microbial community analysis, cultural relics preservation

## Abstract

The Nanhai No. 1 shipwreck is an ancient wooden ship in the Southern Song Dynasty. Currently, serious challenges of microbial diseases exist on the hull wood. This study aimed to obtain microbial samples from the ship hull in December 2021 and analyze the microbial diseases through scanning electron microscopy and high-throughput sequencing to preserve the Nanhai No. 1 shipwreck. The biodegradation mechanism of diseased microorganisms was explored through whole genome sequencing and the detection of enzyme activity and gene expression levels of diseased microorganisms under different conditions. The results showed that there was obvious fungal colonization on the surface of the hull wood and *Fusarium solani* NK-NH1 was the dominant disease fungus on the surface. NK-NH1 has strong cellulose and lignin degradation ability. Its whole genome size is 52,389,955 bp, and it contains 17,402 genes. It has a variety of key enzyme genes involved in cellulose and lignin degradation. The NK-NH1 dominant degrading enzyme lignin peroxidase has the highest enzyme activity at pH = 4, NaCl concentration of 30%, and FeSO_4_ concentration of 50 mg/L, while laccase has the highest enzyme activity at pH = 4, NaCl concentration of 10%, and FeSO_4_ concentration of 100 mg/L. The above research results prove that NK-NH1 is a key fungus to the biodegradation of ship hull wood when it is exposed to air, low pH, high salt, and rich in sulfur iron compounds. This study provides a theoretical basis for the preservation of the Nanhai No. 1 shipwreck.

## 1 Introduction

Cultural relics’ biological corrosion is caused by biological activities, mainly including microorganisms such as bacteria, fungi, and algae, as well as insects such as moths and beetles. The rapid growth of organisms and secretion of secondary metabolites are the most important reasons for the destruction of cultural relics, which may cause irreversible damage. Among them, microbial corrosion, mainly caused by bacteria and fungi, is a recent research focus in cultural relic preservation. Cultural relics of various materials may be corroded by microorganisms (Mazzoli et al., 2018), including organic cultural relics such as wooden relics (Liu et al., 2017), textiles (Gutarowska et al., 2017), silk (Wang et al., 2023), and leather (Liu et al., 2018a). It also includes inorganic cultural relics, such as stone relics (Li et al., 2018), earth ruins (Sun et al., 2020), ancient buildings (Piñar et al., 2009; Gaylarde et al., 2011), and murals (Ma et al., 2020). In particular, organic cultural relics, whose material composition is natural and complex, can provide rich nutrients for microorganism’s growth (Kip and Veen, 2015; Liu et al., 2017). Factors such as airflow and human activities can lead to the deposition and colonization of a large number of microorganisms on the surface of cultural relics, causing changes in the physical and chemical properties of cultural relics, ultimately leading to their damage (Singh, 2012). Bacteria can secrete a variety of secondary metabolites to corrode cultural relics. The hyphae and spores of fungi and actinomycetes will spread and grow on the surface of cultural relics, affecting their artistic and aesthetic value. Simultaneously, they will also invade the interiors of the material, causing damage to the structure of the cultural relic material (Diaz-Herraiz et al., 2013; Mazzoli et al., 2018).

Marine effluent wooden cultural relics are a typical organic matter cultural relic, mainly carried by wooden sunken ships, which are important cultural relics with large volumes, severe corrosion, and difficult preservation. Owing to long-term immersion in seawater, they all have the following characteristics: (1) The moisture content of wood is extremely high; (2) The wood has a high degree of decay, serious corrosion and degradation, and the content of cellulose and hemicellulose in wood is far lower than that of fresh wood; (3) Wood contains high salt content, including soluble salts dominated by sodium chloride and insoluble salts dominated by sulfur iron compounds. High concentrations of salt will promote the degradation and fracture of cellulose in crystallization and dissolution; (4) Ships are often equipped with iron tools such as nails and pans. Sulfate-reducing bacteria and iron tools on the sea floor will form sulfur iron compounds and penetrate wood under long-term action. Sulfur iron compounds will oxidize to generate sulfuric acid and sulfate under the action of oxygen, leading to wood acidification (Fors, 2008; Zhang et al., 2014). Therefore, it is extremely difficult to preserve wooden artifacts in marine water. Famous wooden artifacts from marine water include the Swedish “Vasa” sunken ship (Hocker, 2006), the British “Mary Rose” sunken ship (Preston et al., 2014), the Australian “Batavia” sunken ship, the Chinese “Xiaobaijiao I,” and the Chinese “Huaguangjiao I,” among others. The salvage methods of these sunken ships and the conservation status are different. However, after the sunken ships were salvaged and brought out of the seawater, microbial corrosion needed to be highly valued.

The Nanhai No. 1 shipwreck was an ancient wooden ship that was wrecked and sunk during the Southern Song Dynasty (1127–1279 AD) when it was transporting porcelain out of the Maritime Silk Road. It is a shining pearl on the Maritime Silk Road, key evidence of the ancient Maritime Silk Road, and has critical significance and value for studying marine wood relics (Ma et al., 2022). Among all the cultural relics of the Nanhai No. 1 shipwreck, the wooden hull relic is the most precious and difficult to protect. This is owing to the huge volume and uneven degree of decay of wooden hulls, the long conservation period, and continuous excavation. The average moisture content of the Nanhai No. 1 shipwreck hull wood is 300–700%, which belongs to moderate and severe corrosion. The pH value of the hull wood is stable at 5–7, indicating a neutral to slightly acidic nature. The hull wood contains 6.07–46.13% cellulose, 0.95–8.08% hemicellulose, and 2.63–6.55% ash. Compared with 75% holocellulose content (including cellulose and hemicellulose) and less than 1% ash content in fresh pine (Passialis, 1997), the holocellulose content in wood is far lower than that in fresh wood, indicating that the wood has a high degree of decay and high salt content in wood.

Our research group has been involved in the scientific and technological preservation project of the Nanhai No. 1 shipwreck since April 2015, mainly researching the microbial diseases of the Nanhai No. 1 shipwreck. The preliminary research results show that the dominant disease fungus on the ship is *Fusarium solani* NK-NH1, which has a strong ability to degrade wood (Liu et al., 2018b; Han et al., 2021). Based on previous research, this study comprehensively analyzes the microbial diseases of the Nanhai No. 1 shipwreck by combining traditional microbial separation and detection techniques with cutting-edge sequencing techniques. Simultaneously, the entire genome of the dominant disease fungus *F. solani* NK-NH1 was sequenced, with a focus on studying its biodegradation mechanism. This study has the characteristic of interdisciplinary integration, integrating biology, chemistry, cultural relic protection and other disciplines, reflecting the research characteristics of common orientation and cross-integration. This study provides certain guiding suggestions for the future scientific and technological conservation work of the Nanhai No. 1 shipwreck. Simultaneously, it also provides a reference for the study of microbial diseases in cultural relics and a theoretical basis and data support for the long-term conservation of wooden cultural relics.

## 2 Materials and methods

### 2.1 Investigation and sample collection of microbial diseases on the Nanhai No. 1 shipwreck

**Figure 1:**
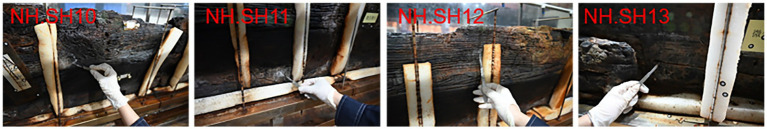
Sampling of microbial diseases.

This study investigated the microbial diseases of the Nanhai No. 1 shipwreck in December 2021. White microbial disease plaques were detected on some areas of the hull surface, mainly in the blind area of the antibacterial agent spraying system. In December 2021, four sampling sites (NH.SH10–NH.SH13) on the hull surface were collected. White plaques were on the surface of these four sampling sites (Figure 1). The sampling sites were all on the deck of Nanhai No. 1 Shipwreck, which was placed near the ship. The spraying system could only spray on some areas of the hull, while the sampling sites could not be sprayed. The average annual temperature of the preservation environment was 25.6°C and average annual humidity was 84.1%.

### 2.2 Scanning electron microscope (SEM) observation

Carbon conductive adhesive was used to adhere microbial samples to the surface of the Nanhai No. 1 shipwreck, which were then dried in a drying dish. After drying, the sample was affixed to the SEM sample stage. Gold was sprayed with a 24-mA current for 300 s, the sample was observed using SEM, and images were recorded. The measurement conditions were as follows: EHT: 15.0 kV, WD: 9.6–10.2 mm, Mag: 0.3KX-5KX.

### 2.3 High throughput sequencing analysis

The DNeasy PowerSoil Pro Kit (QIAGEN, Germany, Cat. No. 47014) was used to extract total DNA from solid samples. The total extracted DNA from samples collected was sent to NovoMagic Technology Co., Ltd. The ITS rRNA genes of distinct regions (ITS1-5F) were amplified using specific primers (ITS5-1737F, ITS2-2043R) with barcodes. Sequencing libraries were prepared with the TruSeq^®^ DNA PCR-Free Sample Preparation Kit (Illumina, United States, Cat. No. 15032317) following the manufacturer’s instructions, and index codes were added. Library quality was assessed using a Qubit@ 2.0 Fluorometer (Thermo Scientific) and an Agilent Bioanalyzer 2,100 system. The library was then sequenced on an Illumina NovaSeq platform, generating 250 bp paired-end reads. Paired-end reads were matched to samples based on their unique barcodes and then trimmed to remove the barcodes and primer sequences. The paired-end reads were merged using FLASH (V1.2.7, http://ccb.jhu.edu/software/FLASH). Quality filtering of raw tags was done under specific conditions to obtain high-quality clean tags following the QIIME (V1.9.1, http://qiime.org/scripts/split_libraries_fastq.html) quality control process. The tags were compared to the Silva database using the UCHIME algorithm^1^ to identify and remove chimeric sequences. The effective tags were then obtained. Sequence analysis was conducted using the Uparse software (Uparse v7.0.1001, http://drive5.com/uparse/). Sequences with ≥97% similarity were assigned to the same operational OTUs. A representative sequence from each OTU was selected for further annotation, using the Silva Database^2^ and the Mothur algorithm to annotate the taxonomic information. The abundance information of OTUs was normalized to a standard sequence number corresponding to the sample with the lowest number of sequences. The raw sequencing data are accessible at the NCBI Sequence Read Archive (SRA) with the study accession number PRJNA1015580.

### 2.4 Fungal whole genome sequencing analysis

NK-NH1 was cultured in a PD liquid medium at 28°C for 3 days. It was centrifuged at 4°C using a 50 mL centrifuge tube at low speed, rinsed with distilled water three times, and the fungal body was collected through centrifugation. It was frozen in liquid nitrogen and sent to BGI Gene (China). The data volume of fungal whole genome sequencing is large and the parsing is complex. Currently, a combination of short-read long and single molecule sequencing is used to achieve *de novo* assembly of fungal genome fine maps. The raw sequencing data are accessible at the NCBI WGS with the study accession number PRJNA1016167.

### 2.5 Detection of the activity and specific activity of cellulose and lignin-degrading enzymes in microorganisms

The improved Lowry protein quantification kit was used to measure the total protein content in the supernatant, and then measure the activities of the corresponding enzymes according to the following method.

#### 2.5.1 Detection of cellulase and specific activities

##### 2.5.1.1 Preparation of glucose standard curve

Multiple gradient glucose solutions were prepared. Approximately 1 mL of 1% CM-Na buffer solution was taken, soaked in 50°C water for 30 min, 2 mL of DNS was added, followed by 0.5 mL of glucose gradient solution. It was boiled for 10 min, cooled, and the absorbance at 550 nm was measured. A standard curve was drawn with glucose concentration as the horizontal axis and absorbance value as the vertical axis.

##### 2.5.1.2 Determination of glucose concentration in the supernatant

The experimental group consisted of taking 0.5 mL of the supernatant, adding 1 mL of 1% CMC-Na buffer, bathing at 50°C for 30 min, adding 2 mL of DNS, and boiling for 10 min. The blank control was prepared by taking 1 mL of 1% CMC-Na buffer solution, water bathing at 50°C for 30 min, adding 2 mL of DNS, adding 0.5 mL of supernatant, and boiling for 10 min. The absorbance at 550 nm was measured after cooling in a cold-water bath. Finally, the glucose concentration in the supernatant was obtained based on the glucose standard curve.

##### 2.5.1.3 Determination of cellulase and specific activities

The unit of cellulase activity (U) is defined as the amount of enzyme required to produce 1 mmol of glucose per minute. The enzyme activity unit U/mL per milliliter of enzyme solution = (X × V1)/(M × T × V2); the specific activity of cellulase U/ng = (X × V1)/(M × T × V2 × P). In the above equation, *X* is the glucose concentration (mg/mL) calculated based on the standard curve; V1 is the volume of the reaction solution (mL); *M* is the molar mass of glucose; *T* is the reaction time (min); V2 is the volume of the supernatant (mL); and *P* is the total protein concentration (ng/mL) calculated based on the standard curve.

#### 2.5.2 LiP activity and specific activity detection

Approximately 1 mL of 125 mmol/L tartaric acid sodium buffer solution was taken, 0.5 mL of 0.16 mmol/L aniline blue solution, 0.5 mL of supernatant, and 0.5 mL of 2 mmol/L H_2_O_2_ solutions were added to start the reaction. The absorbance change value at 651 nm was determined in the first 3 min. The LiP activity unit (U) is defined as the amount of enzyme required to decrease the OD value by 0.1 per min. The enzyme activity unit per milliliter of enzyme solution (U/mL) = N/(0.1 × T × V2). Specific activity of LiP (U/ng) = N/(0.1 × T × V2 × P). In the above equation, *N* is the absorbance change value at 651 nm in the first 3 min; T is the reaction time (min); V2 is the volume of the supernatant (mL); and *P* is the total protein concentration (ng/mL) calculated based on the standard curve.

#### 2.5.3 MnP activity and specific activity detection

Approximately 3.4 mL of 50 mmol/L sodium lactate buffer and 0.1 mL of 1.6 mmol/L MnSO_4_ solutions were taken, 0.4 mL of supernatant was added, and preheated at 37°C for 10 min. Following this, 0.1 mL of 1.6 mmol/L H_2_O_2_ solution was added and the absorbance change at 240 nm was measured in the first 3 min. The MnP activity unit (U) is defined as the amount of enzyme required to increase the OD value by 0.1 per min. The enzyme activity unit per milliliter of enzyme solution (IU/mL) = N/(0.1 × T × V2). Specific activity of MnP (U/ng) = N/(0.1 × T × V2× P). In the above equation, *N* is the absorbance change value at 240 nm in the first 3 min; *T* is the reaction time (min); V2 is the volume of the supernatant (mL); and *P* is the total protein concentration (ng/mL) calculated based on the standard curve.

#### 2.5.4 Lac activity and specific activity detection

Approximately 3 mL of 200 mmol/L acetic acid buffer was taken, 0.5 mL of 7 mmol/L ABTS solution and 0.5 mL of supernatant were added, and the absorbance change at 420 nm was measured in the first 3 min. The Lac activity unit (U) is defined as the amount of enzyme required to increase the OD value by 0.01 per min. The enzyme activity units contained in each milliliter of enzyme solution (IU/mL) = N/(0.01 × T × V2). Specific activity of Lac (U/ng) = N/(0.01 × T × V2 × P). In the above equation, *N* is the absorbance change value at 420 nm in the first 3 min; *T* is the reaction time (min); V2 is the volume of the supernatant (mL); and *P* is the total protein concentration (ng/mL) calculated based on the standard curve.

### 2.6 Detection of gene expression levels of dominant degrading enzymes under different culture conditions using qPCR

#### 2.6.1 Setting of different cultivation conditions

Based on the hull wood powder liquid culture medium, four single variables were set successively:

##### 2.6.1.1 The pH value

A pH gradient was set to detect the activity and specific activity of dominant enzymes and expression level of enzyme genes, to obtain the optimal pH value.

##### 2.6.1.2 NaCl concentration

A NaCl concentration gradient was set to detect the activity and specific activity of dominant enzymes, as well as the expression of enzyme genes, under the optimal pH conditions to obtain the optimal NaCl concentration.

##### 2.6.1.3 FeSO_4_ concentration

The FeSO_4_ concentration gradient was set to detect the activity, specific activity, and expression level of dominant degrading enzymes under the optimal pH value and NaCl concentration conditions, respectively, to obtain the optimal FeSO_4_ concentration.

##### 2.6.1.4 PEG400 concentration

The PEG400 concentration gradient was set to detect the activity, specific activity, and expression level of dominant degrading enzymes under the optimal pH value, NaCl concentration, and FeSO_4_ concentration conditions, respectively, to obtain the optimal PEG400 concentration.

#### 2.6.2 qPCR primer design

The data obtained from whole gene sequencing was analyzed, and combined with relevant literature reports, and Primer 5 software was used to design qPCR primers.

#### 2.6.3 Trizol method for extracting fungal RNA

The culture precipitate was collected by centrifugation at 4°C, ground with liquid nitrogen, and then an appropriate amount of dry powder was taken into a 1.5 mL EP tube. Approximately 1 mL of Trizol was added to the EP tube and shaken for 2 min. Approximately 200 μL chloroform was added, left to stand for 5 min, centrifuged, and the supernatant was aspirated into a new EP tube. Approximately 500 μL isopropanol was added, left to stand at −20°C for 10 min, centrifuged, and the supernatant was discarded. Approximately 500 μL 70% ethanol was added, centrifuged, the supernatant was discarded, and dried for 5 min. For the preparation of the digestive DNA reaction system (RNA, 10× DNase I buffer, recombinant DNase I, RNase inhibitor, RNase-free H_2_O), a total of 50 μL reacts in a 37°C incubator for 20–30 min. Approximately 50 μL RNase-free H_2_O and 100 μL phenol/chloroform/Isoamyl alcohol (25:24:1) were added and completely mixed. After centrifugation, the upper layer was taken to a new EP tube and same amount of chloroform/isoamyl alcohol (24:1) was added. After centrifugation, the upper layer was transferred to a new EP tube. NaAC and anhydrous ethanol were added and left at −80°C for 20 min. The supernatant was discarded after centrifugation. Approximately 500 μL 75% ethanol was added, centrifuged, and the supernatant was discarded. After drying, it was dissolved with an appropriate amount of RNase-free H_2_O for RNA reverse transcription.

#### 2.6.4 RNA reverse transcription to obtain cDNA

The template RNA/primer system was prepared in a PCR tube [template RNA 2 μg, Oligo (dT) 2 μL]. RNase-free H_2_O replenishment was performed to 12 μL. After 10 min at 70°C, it was quickly placed on ice for 3 min. A reaction system was prepared in the PCR tube (system mentioned above 12 μL, buffer 4 μL, dNTP mixture 1 μL, RNase inhibitor 0.5 μL. RTase 1 μL, and RNase-free H_2_O 1.5 μL) 20 μL in total. It was held at 42°C for 1 h, cooled at 70°C for 15 min on ice, and the obtained cDNA was used for qPCR detection of enzyme gene expression.

#### 2.6.5 qPCR detection of enzyme gene expression levels

The qPCR reaction system comprised cDNA 1 μL, primers each 0.5 μL, SYBR 10 μL, and RNase-free H_2_O 8 μL. The prepared reaction system was placed on ice and stored away from light. The following program was set on the qPCR instrument for gene amplification. Finally, the *C*t values during the amplification process were obtained and expression levels of different groups were compared. Three parallel samples were prepared for each sample.

### 2.7 Statistical analysis

This research was carried out in triplicate with completely randomized experimental designs. Data obtained in this investigation were depicted as averages ± standard deviation. The statistical significance between the results of different sample groups were analyzed by *T*-test. Statistical significance was set at *p* < 0.05, ^*^p < 0.05, ^**^p < 0.01, ^***^p < 0.001.

## 3 Results

### 3.1 SEM observation and high-throughput sequencing analysis of microorganisms on the hull of the Nanhai No. 1 shipwreck in December 2021

**Figure 2:**
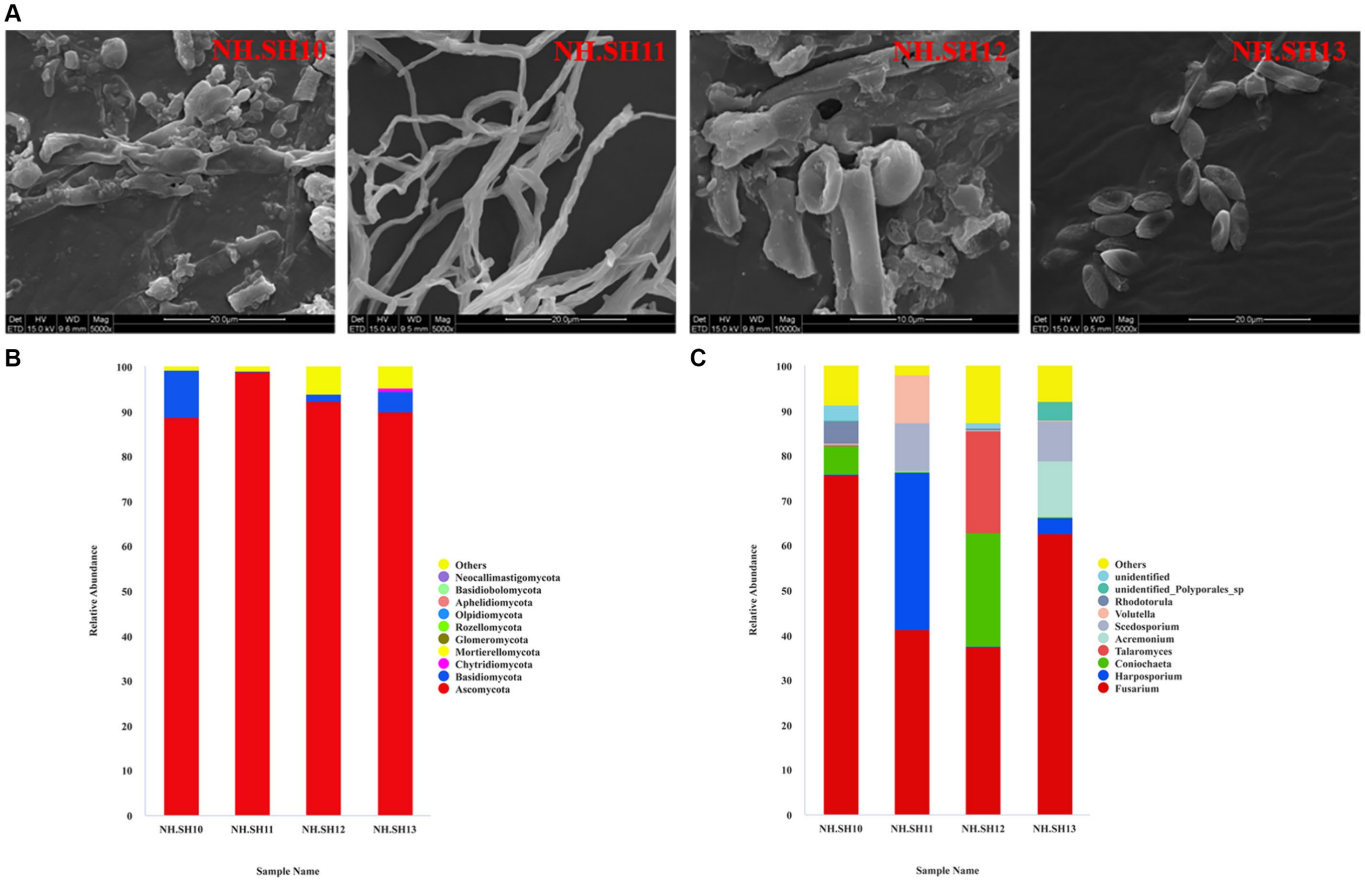
Composition analysis of fungal community on the hull of the Nanhai No. 1 shipwreck (December 2021). **(A)** SEM observation results; **(B)** relative abundance of fungi at the phylum level; **(C)** relative abundance of fungi at the genus level.

In December 2021, microbial disease samples were collected from four sites (NH.SH10–NH.SH13) on the hull surface. The SEM results indicated that the typical structure of filamentous fungi still adheres to the surface of the ship, with a high abundance (Figure 2A). The high-throughput sequencing results of fungi show that at the phylum level, the dominant phylum was Ascomycota, with an average relative abundance of 92.48% (Figure 2B). At the level of fungi, *Fusarium* accounted for the largest proportion, with an average relative abundance of 54.23%. Additionally, *Harposporium*, *Coniochaeta*, *Talaromyces*, *Acremonium*, and *Scedosporium* were present (Figure 2C). Compared with previous results (Liu et al., 2018b; Han et al., 2021), the dominant disease fungus on the hull surface was *Fusarium* since 2015.

### 3.2 Whole genome sequencing analysis of *F. solani* NK-NH1

#### 3.2.1 Genomic component analysis

**Figure 3:**
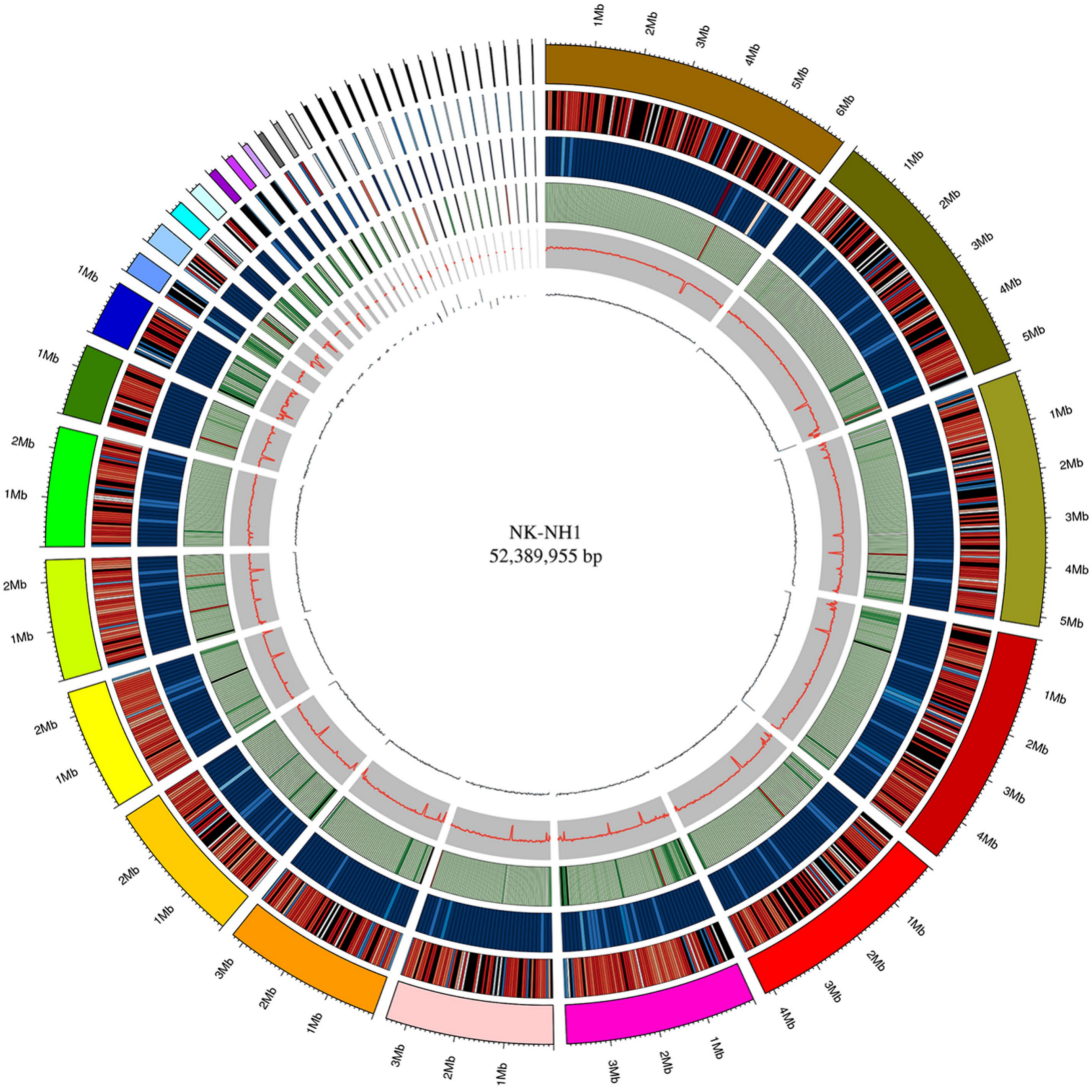
Whole genome circle graph of *F. solani* NK-NH1. The circle graph from outside to inside is as follows: (1) genome (sorted by length); (2) gene density (number of genes in 50 kp non-overlapping window); (3) non-coding RNA density (number of non-coding RNA in 100 kp non-overlapping window); (4) repeat sequence (repeat sequence in 50 kp non-overlapping window); (5) GC (GC rate in 20 kp non-overlapping window); (6) GC skew (GC offset in 20 kp non-overlapping window).

The whole genome size of *F. solani* NK-NH1 is 52,389,955 bp and contains 17,402 genes. The total length of the CDS sequence is 25,625,398 bp, accounting for 48.91% of the total genome length. Non-coding RNAs (including tRNA, rRNA, sRNA, snRNA, and miRNA) collectively account for 0.1534% of the genome. Repetitive sequences (including DNA transposable elements, tandem repeats, and transposable elements) account for 7.2366% of the genome. According to the genome sequence of NK-NH1, Circos software is used to analyze the genome, gene density, non-coding RNA, repeat sequence, GC-content, GC skew, and other information, and draw the whole genome circle of NK-NH1 (Figure 3).

#### 3.2.2 Gene function annotation analysis

**Figure 4:**
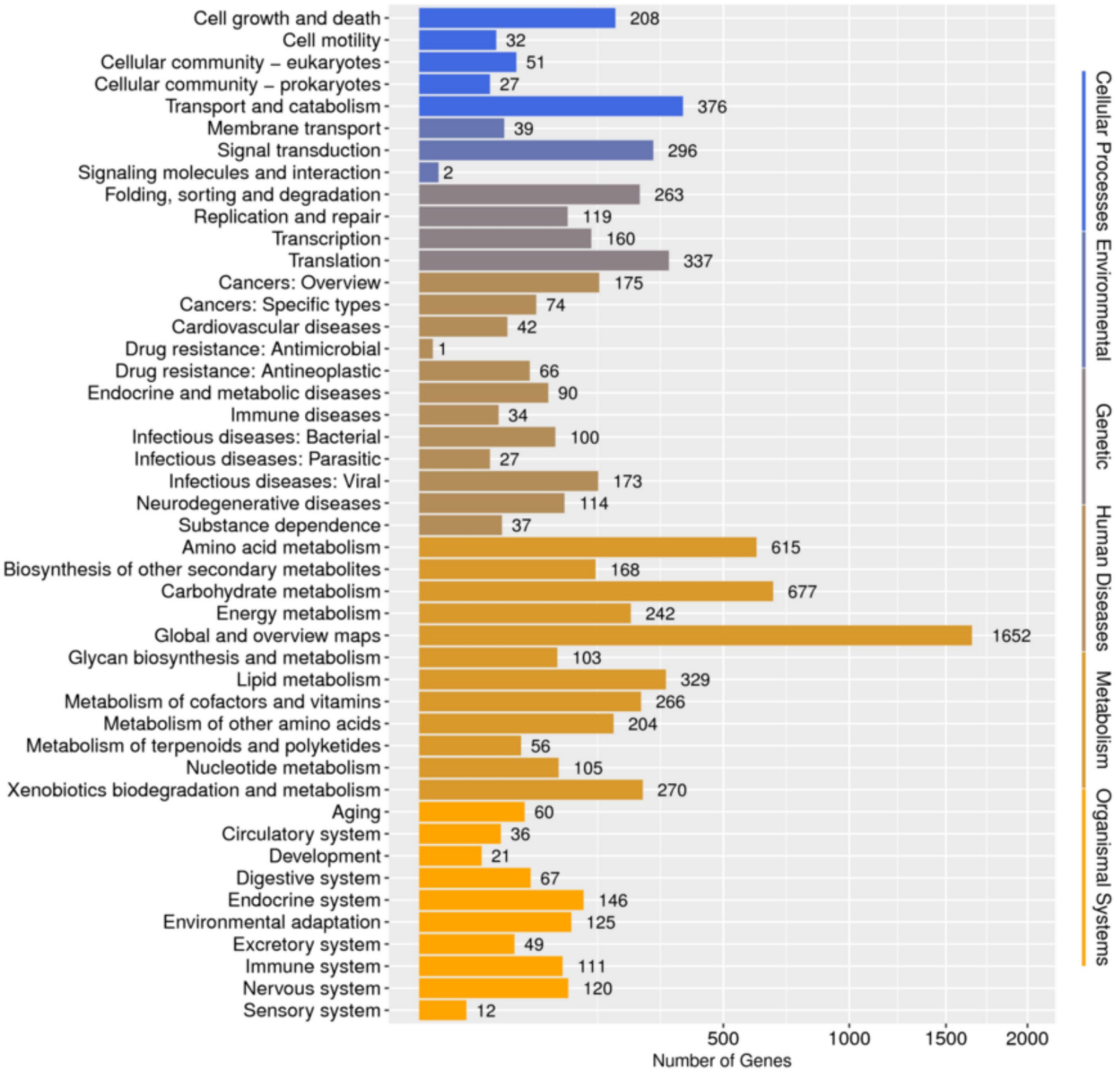
Functional annotation distribution of secondary classification of KEGG database in the whole genome analysis of *F. solani* NK-NH1.

**Figure 5:**
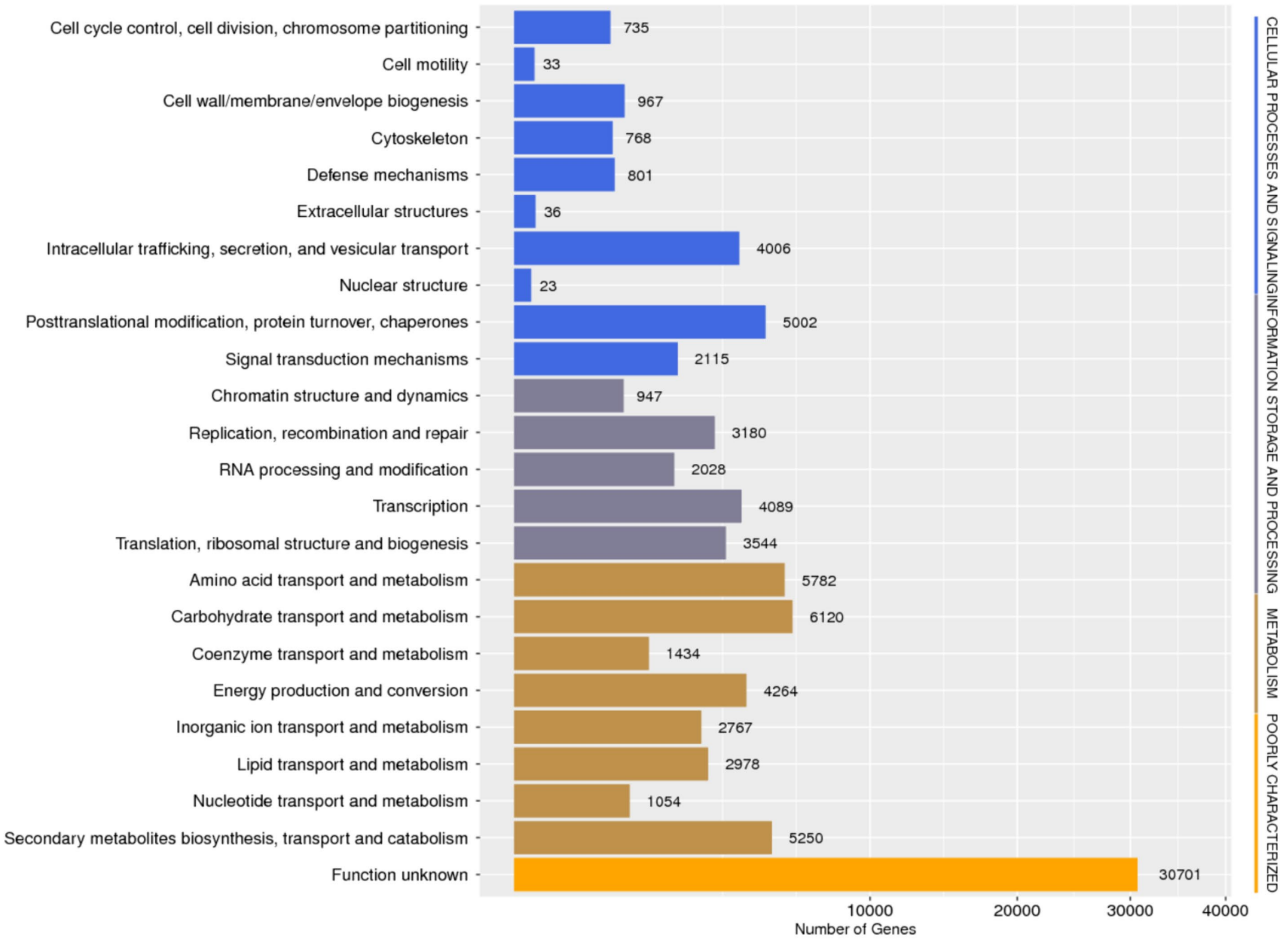
Functional annotation distribution of eggNOG database in the whole genome analysis of *F. solani* NK-NH1.

Database comparison and annotation were conducted on the entire genome of *F. solani* NK-NH1. A total of 16 databases were annotated, with a total of 16,950 (97.4%) genes annotated. The statistical results are shown in Supplementary Table S1. An analysis was conducted on the annotation of CAZy, eggNOG, and KEGG databases, and the results showed that carbohydrate metabolism accounted for a large proportion of 12.32% in the secondary classification of KEGG databases (Figure 4). The proportion of carbohydrate transport and metabolism annotations obtained in the functional annotations of the eggNOG database is the highest, reaching 48.47% (Figure 5). Among all the gene functional annotations, the genes related to the cellulose and lignin degradation ability of NK-NH1 were explored. After extensive data screening, they were mainly found to be annotated in CWDE, KEGG, NR, eggNOG, GO, Swiss-Prot, KOG, and CAZy databases. The key genes annotated are shown in Table 1.

**Table 1:** Key genes of cellulose and lignin degradation in the whole genome sequencing of *F. solani* NK-NH1.

Database	Key genes of cellulose and lignin degradation
CWDE	Beta-glucosidase (GME9790), endoglucanase (GME7240), endo-beta-1,4-glucanase (GME2370), cellulose 1,4-beta-cellobiosidase (GME10478)
KEGG	Manganese peroxidase (GME5842), endo-beta-1,4-glucanase (GME14830), endoglucanase (GME12687), beta-glucosidase (GME14573), endo-1,3(4)-beta-glucanase (GME4480)
NR	Beta-glucosidase (GME10767), exoglucanase (GME1814)
eggNOG	Endoglucanase (GME1127)
GO	Manganese superoxide dismutase (GME3578), fungal lignin peroxidase (GME5842), beta-glucosidase (GME1026), endo-1,3(4)-beta-glucanase (GME4480), endoglucanase (GME15228),
Swiss-Prot	Laccase (GME3721), endoglucanase (GME15228), xyloglucanase (GME12458), exoglucanase (GME14114), glucosidase (GME10300), beta-glucosidase (GME1026), endo-beta-1,4-glucanase (GME14830), endo-1,6-beta-glucosidase (GME9361)
KOG	Glucosidase (GME10300), beta-glucosidase (GME1026)
CAZy	Beta-glucosidase (GME1026), laccase (GME11713)

### 3.3 Effects of different culture conditions on enzyme activity of *F. solani* NK-NH1

**Figure 6:**
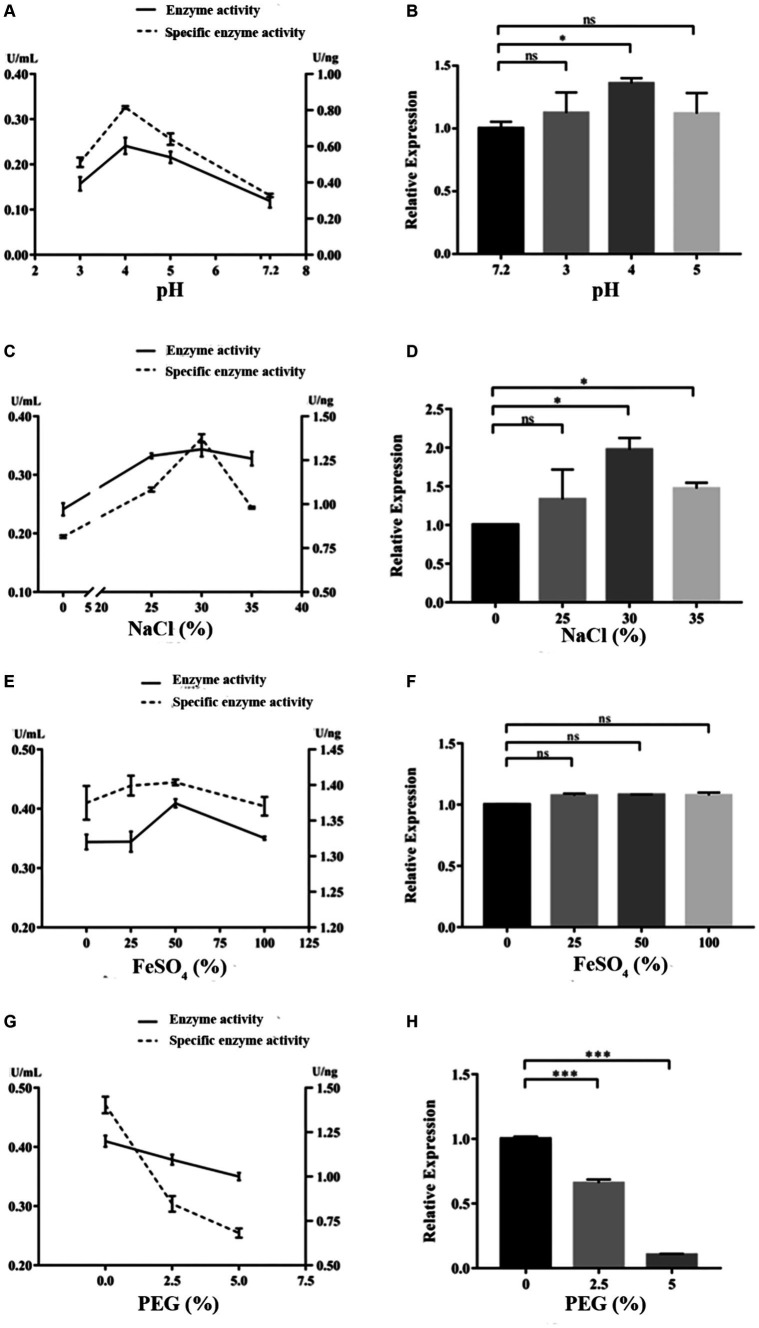
The variation trend of LiP enzyme activity, specific activity, and enzyme gene expression of *F. solani* NK-NH1 under different culture conditions. **(A)** Effects of pH on LiP enzyme and specific activities. **(B)** Effect of pH on LiP enzyme gene expression. **(C)** Effect of NaCl concentration on LiP enzyme and specific activities. **(D)** Effect of NaCl concentration on LiP enzyme gene expression. **(E)** Effect of FeSO_4_ concentration on LiP enzyme and specific activities. **(F)** Effect of FeSO_4_ concentration on LiP enzyme gene expression. **(G)** Effect of PEG400 concentration on LiP enzyme and specific activities. **(H)** Effect of PEG400 concentration on LiP enzyme gene expression. Statistical significance was set at *p* < 0.05, *p < 0.05,**p < 0.01, ***p < 0.001.

**Figure 7:**
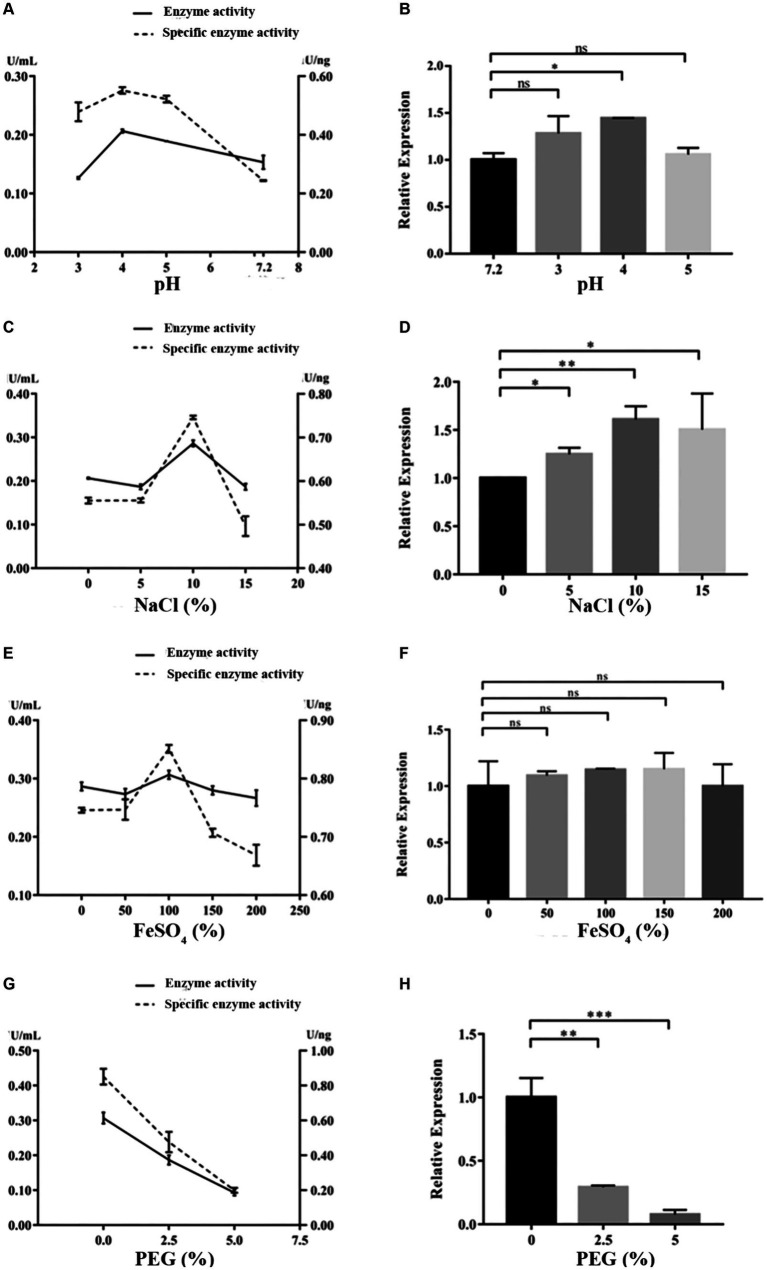
The variation trend of Lac enzyme activity, specific activity, and enzyme gene expression of *F. solani* NK-NH1 under different culture conditions. **(A)** Effect of pH on Lac enzyme and specific activities. **(B)** Effect of pH on Lac enzyme gene expression. **(C)** Effect of NaCl concentration on Lac enzyme and specific activities. **(D)** Effect of NaCl concentration on Lac enzyme gene expression. **(E)** Effect of FeSO_4_ concentration on Lac enzyme and specific activities. **(F)** Effect of FeSO_4_ concentration on Lac enzyme gene expression. **(G)** Effect of PEG400 concentration on Lac enzyme and specific activities. **(H)** Effect of PEG400 concentration on Lac enzyme gene expression. Statistical significance was set at *p* < 0.05, *p < 0.05,**p < 0.01, ***p < 0.001.

Under the cultivation conditions of using the experimental wood powder of the Nanhai No. 1 shipwreck as the sole carbon source, the dominant degrading enzymes of NK-NH1 are LiP and Lac, with the highest enzyme activity occurring for 8.5 days (Supplementary Table S2). Currently, the Nanhai No. 1 shipwreck is in an acidic, high salt, and sulfur-rich iron compounds preservation environment, and archeological workers are using the protective material PEG400 to dewater and strengthen it. By adjusting pH value, and NaCl, FeSO_4_, and PEG400 concentrations, the effects of various factors on the LiP and Lac activity of NK-NH1 were studied, and the reason for NK-NH1 becoming a dominant fungus was explored. The research results found that the highest enzyme activity of LiP of NK-NH1 was generated under the following conditions: pH = 4, NaCl concentration of 30%, and FeSO_4_ concentration of 50 mg/L, without the addition of PEG400 (Figure 6). The maximum enzyme activity of Lac is generated under the following conditions: pH = 4, NaCl concentration of 10%, and FeSO_4_ concentration of 100 mg/L, without the addition of PEG400 (Figure 7). The detection results of enzyme gene expression are consistent with those of enzyme activity detection.

## 4 Discussion

The Nanhai No. 1 shipwreck, as one of the rare large and precious wooden cultural relics in China, has extremely important historical significance and conservation value. The microbial diseases on the hull are the biggest challenge in its conservation. Although the effective antibacterial agent K100 has been regularly and quantitatively sprayed on-site through the installed spraying system, owing to the complex structure of the ship, there are blind spots in the spraying system, and the loss of antibacterial agents exists, resulting in microbial contamination on some ship surfaces.

Fungi are the most effective and abundant wood decomposers known to date. Their developed hyphae give them the advantage of spreading growth over most prokaryotes. Many fungi isolated from wooden artifacts can produce cellulase and lignin-degrading enzymes. For example, *Penicillium* and *Cladosporium* (Zhang et al., 2019) separated from the canoe of the Tang Dynasty in the National Marine Museum of China, and *Aspergillus* and *Penicillium* (Osman et al., 2015) separated from the Islamic Art Museum and other cultural relics. *Fusarium* can be widely distributed in various environments and can degrade wood, significantly damaging wood (Rodriguez et al., 1996; Panagiotou et al., 2003; M’Barek et al., 2019). There is limited research on the mechanism of wood degradation by *Fusarium*; however, *F. solani* is currently known to degrade cellulose and lignin, producing cellulase, Lac, LiP, and MnP (Saparrat et al., 2000; Wu et al., 2010; Obruca et al., 2012). After being infected with *F. solani*, the cellulose and hemicellulose content in wood will decrease (Rahmawati et al., 2020). Benaddou et al. (2024) experiment revealed that *F. oxysporum* and *F. solani* were the most selective lignin-degrading fungi among the tested fungi. The cell wall degrading enzyme (CWDE) in *Fusarium* is the main pathogenic factor for its invasion and infection of plants, mainly including pectinase, cellulase, and protease (Erazo et al., 2021). Among numerous CWDE, the deletion of a single gene does not significantly affect the virulence of *Fusarium*; however, if two or more coding genes are knocked out simultaneously and an upstream gene regulating CWDE is knocked out, the pathogenicity will be weakened (Ruiz et al., 2016; Paccanaro et al., 2017). *F. solani* can decompose aromatic acids (Norris, 1980), and the CWDE produced during spore germination can enhance its infectivity (Köller et al., 1982). Currently, *F. solani* NK-NH1 has colonized the Nanhai No. 1 shipwreck surface and has become a dominant fungus. This study sequenced its entire genome, and through database annotation, it was found that genes related to carbohydrate transport and metabolism account for a large proportion in various major databases, and multiple key genes involved in cellulose and lignin degradation have been compared in various databases.

The hull wood of the Nanhai No. 1 shipwreck has the characteristics of weak acidity, high salinity, and high sulfur iron compounds, and the surface has the protective material PEG. pH is a critical element that affects enzymatic reactions because it regulates the ionization state of acidic or basic amino acids on the enzyme site and/or affinity to substrate (Xu, 1997). It was reported that NaCl at relatively low concentrations can bind active sites to enhance the affinity of enzyme to substrate and facilitate oxygen reduction (Si et al., 2021a). Additionally, some enzymes require metallic ions to retain the conformation of their active sites (Si et al., 2021b). Therefore, we tested the impact of these factors on NK-NH1 and found that under the cultivation condition of experimental shipwreck wood powder as the only carbon source, the highest enzyme activity of the dominant degrading enzyme LiP was produced under the following conditions: pH = 4, NaCl concentration 30%, FeSO_4_ concentration 50 mg/L, and no PEG400 added. The highest enzyme activity conditions for the dominant degrading enzyme Lac are pH = 4, NaCl concentration of 10%, FeSO_4_ concentration of 100 mg/L, and no PEG400 added. This result indicates that the current state of the hull with low pH, high salt, and rich sulfur iron compounds is conducive to the degradation of wood by *F. solani* NK-NH1. This also explains from another perspective the dominant colonization of NK-NH1 on the hull of the Nanhai No. 1 shipwreck. This is consistent with some previous literature reports (Barclay et al., 1998; Firdous and Shahzad, 2001; Mandeel, 2006; Merlin et al., 2013; Zheng et al., 2017; Dutta et al., 2018).

In addition to cellulose and lignin-degrading enzymes, fungi can also accelerate the degradation of cellulose and lignin through the Fenton reaction (Baldrian and Valášková, 2008). Fe^2+^, H_2_O_2_, and acidic conditions are necessary conditions for the occurrence of Fenton reaction. There are multiple metabolic pathways in organisms that can produce H_2_O_2_. The hull is rich in sulfur and iron compounds. Therefore, it is highly likely that *F. solani* NK-NH1 will produce a Fenton reaction to degrade the hull wood. Through whole genome sequencing, key genes that may be involved in sulfur and iron metabolism in NK-NH1 are found, which are mainly annotated in NR, GO-IPR, KOG, KEGG, NOG, GO, TCDB, and Swiss-Prot databases (Supplementary Table S3 illustrates the key genes annotated). It mainly includes cysteine desulfurizing enzyme, sulfite oxidase, FeS cluster-related protein, sulfite transport and efflux-related protein, and thiosulfate sulfur transferase.

In summary, the current dominant disease fungus, *F. solani* NK-NH1 promoted the degradation of hull wood. Moreover, the existing low pH, high salt, and sulfur-rich iron compounds preservation state of the Nanhai No. 1 shipwreck will, to a certain extent, promote its degradation of hull wood. Therefore, regular monitoring, physical control, and biochemical antibacterial methods must be combined to address the microbial diseases of the Nanhai No. 1 shipwreck.

## 5 Conclusion

This study analyzed the microbial diseases of the Nanhai No. 1 shipwreck and biodegradation mechanism of the dominant fungus. The high-throughput sequencing results showed that *Fusarium* is the dominant fungus, and whole genome sequencing results of the isolated *F. solani* NK-NH1 showed that it can degrade wood. The highest enzyme activity of the NK-NH1 dominant degrading enzyme LiP is generated under conditions of pH = 4, NaCl concentration of 30%, FeSO_4_ concentration of 50 mg/L, and no addition of PEG400. The highest enzyme activity of Lac is generated under conditions of pH = 4, NaCl concentration of 10%, FeSO_4_ concentration of 100 mg/L, and no PEG400 added. This indicates that the current preservation state of the hull with low pH, high salt, and sulfur-rich iron compounds is conducive to the biodegradation of hull wood by *F. solani* NK-NH1.

## Data availability statement

The datasets presented in this study can be found in online repositories. The names of the repository/repositories and accession number(s) can be found in the article/Supplementary material.

## Author contributions

YW: Formal analysis, Investigation, Software, Writing – original draft, Writing – review & editing. YH: Investigation, Methodology, Writing – original draft. NL: Investigation, Writing – original draft. CW: Writing – original draft. KM: Writing – original draft. XH: Writing – original draft. JD: Supervision, Writing – original draft. HG: Supervision, Validation, Visualization, Writing – review & editing. JP: Conceptualization, Funding acquisition, Supervision, Validation, Visualization, Writing – review & editing.

## Footnotes

^1^ http://www.drive5.com/usearch/manual/uchime_algo.html

^2^ http://www.arb-silva.de
