# Supplementary material for: Study on biodegradation mechanism of Fusarium solani NK-NH1 on the hull wood of the Nanhai No. 1 shipwreck
Source: Front Microbiol. 2024 May 30;15:1382653. doi: 10.3389/fmicb.2024.1382653 (PMC11173092; doi:10.3389/fmicb.2024.1382653)
Supplement: Supplementary file 1 [file Table_1.DOCX]

Table S1. Statistical results of whole genome functional annotations of NK-NH1

| Database | Number and proportion of annotated genes |
| --- | --- |
| P450 | 3,439（19.76%） |
| TF | 1,019（5.85%） |
| CAZy | 611（3.51%） |
| IPR | 13,215（75.93%） |
| KINASE | 164（0.94%） |
| Swissprot | 4,145（23.81%） |
| eggNOG | 12,625（72.54%） |
| CARD | 0（0%） |
| CWDE | 124（0.71%） |
| NR | 16,810（96.59%） |
| GO | 9,322（53.56%） |
| KEGG | 5,495（31.57%） |
| DBCAN | 808（4.64%） |
| TCDB | 788（4.52%） |
| PHOSPHATASE | 35（0.2%） |
| KOG | 2,623（15.07%） |

Table S2. The highest enzyme activity and production time of cellulose and lignin degradation enzymes of *F. solani* NK-NH1

| Enzyme | Maximum enzyme activity (U/mL) | Cultivation time (days) |
| --- | --- | --- |
| Cellulase | 0.0456 | 9 |
| LiP | 0.1020 | 8.5 |
| MnP | 0.0433 | 7 |
| Lac | 0.1533 | 8.5 |

Table S3. Key genes of sulfur and iron metabolism in the whole genome sequencing of *F. solani* NK-NH1

| Database | Number and proportion of annotated genes |
| --- | --- |
| NR | Cysteine desulfurase（GME11248） |
| GO-IPR | Cysteine desulfurase（GME11248） |
| KOG | Cysteine desulfurase（GME11248） |
| KEGG | Cysteine desulfurase（GME11248）、Sulfite oxidase（GME1519） |
| NOG | Cysteine desulfurase（GME11248）、Sulfite oxidase（GME9767） |
| GO | FeS cluster related proteins（GME10171） |
| TCDB | Sulfite transporter Ssu1（GME851） |
| Swissprot | Sulfite efflux pump SSU1（GME851）、Sulfite oxidase（GME1519）、Thiosulfate sulfurtransferase TUM1（GME14732） |
